# Determining Barriers to Dietary Change Among Medically Underserved Men With Prostate Cancer

**DOI:** 10.1177/10901981261421578

**Published:** 2026-02-25

**Authors:** M. Smith, R. S. Garcia, D. Cho, M. Tariba-Edick, T. Lawen, C. Daniel, L. McNeill, C. Pettaway, J. R. Gregg

**Affiliations:** 1 The University of Texas MD Anderson Cancer Center, Houston, TX, USA

**Keywords:** Mediterranean, prostate cancer, Black and Hispanic, qualitative studies

## Abstract

Prostate cancer (PCa) disproportionately affects Black men and men of low socioeconomic status, who are more likely to experience higher incidence, disease progression, and mortality. Despite this, cardiovascular disease remains a leading cause of death among men with localized disease. Therefore, lifestyle interventions, particularly diet-based, provide an opportunity to drastically improve outcomes. Little is known about how underserved men with PCa and their families perceive barriers to dietary change, including adaptation of the cardioprotective Mediterranean diet (MD). This qualitative study explored dietary knowledge, cultural influences, and willingness to participate in dietary interventions among Black and Hispanic men with localized PCa at a county hospital. Six focus groups were conducted in English or Spanish with 31 total participants (men and their partners) recruited from a safety-net county hospital. Thematic analysis was guided by the health belief model and theory of planned behavior. Four themes emerged: First, the attitudes toward healthy diet and physician advice; second, the positive and negative influence of family, friends, and culture; third, the emotional and spiritual influences on dietary behavior; and finally, structural barriers and their impact on self-efficacy. Findings underscore the critical role of buy-in from family, the importance of education and instruction, and the value of maintaining space for emotion and spirituality. These results support the development of partner-engaged, culturally tailored dietary interventions for medically underserved populations with PCa.

## Implications for Practice

Here, we describe focus groups among Black and Hispanic medically underserved prostate cancer patients, and their spouses, about attitudes and beliefs about diet and dietary change. We describe multiple themes shared by these groups, including attitudes toward diet and physician advice, the influence of family and friends, spiritual and emotional factors, and structural barriers to change. These findings have implications in the design of future Mediterranean diet–based behavioral interventions for medically underserved populations.

Both Black men and men of low socioeconomic status (SES) face disproportionately poor outcomes from prostate cancer (PCa). In the United States, the incidence and age-adjusted mortality is 1.7 and 2.1 times higher in Black men than in non-Hispanic White men, respectively (Siegel et al., 2021). Although this disparity can be partially explained by socioeconomic differences, including access to care, there is compelling evidence that PCa in Black men is more aggressive and that this is one root cause of the greater mortality (Lillard et al., 2022; Powell et al., 2013). Moreover, while Siegel et al. (2021) suggest that incidence and mortality rates are similar for Hispanic men and non-Hispanic White men, Ward et al. (2004) found that low SES increases PCa mortality in men of all races and ethnicities (Ward et al., 2004). In particular, this includes the uninsured, as uninsured men have a PCa mortality rate almost twice that of insured men (Niu et al., 2013).

Given these health disparities, modifiable factors (diet, physical activity) can provide major opportunities for intervention (O’Connor et al., 2020). This is especially relevant for men with low-risk PCa who are managed through active surveillance (AS), a strategy involving regular clinical monitoring for signs of disease progression (Hamdy et al., 2016; Mohler et al., 2019; Resnick et al., 2013). In this population, cardiovascular disease is the leading cause of death, particularly when other health conditions are present (Bill-Axelson et al., 2018). Notably, the behavioral factors mentioned are known to help prevent cardiovascular disease (O’Connor et al., 2020).

Regarding diet, the Mediterranean diet (MD; which is characterized by the intake of legumes, whole grains, vegetables, fruits, fish, nuts, and olive oil) is well known for its anti-inflammatory and lipid-lowering properties (Chryohoou et al., 2004; Gausch-Ferre et al., 2017). The PREDIMED study, a landmark randomized trial by Estruch et al. (2018), demonstrated that behavioral MD interventions decrease cardiovascular events and strokes. In addition, studies indicate that MD may alter risk of PCa progression (Gregg et al., 2021; Kenfield et al., 2014; Russo et al., 2019; Schneider et al., 2019) including limited data from our group that baseline MD adherence may decrease risk of early-stage disease progression, including in non-White men (Gregg et al., 2021).

While the MD shows significant promise, adherence remains a challenge, particularly among Black and Hispanic men. Across studies, barriers to MD adherence include lack of familiarity with Mediterranean foods, resistance to changing eating habits, and concerns that the diet may not align with personal or cultural taste preferences (Hardin-Fanning, 2013; Kretowicz et al., 2018; Moore et al., 2018). However, most of these studies did not include underserved Black and Hispanic participants, limiting the generalizability of these findings.

Separately, there is strong evidence that low-income Black and Hispanic men with PCa face additional barriers to adopting any healthy dietary pattern. Haymer et al. (2020) showed that low-income men with PCa have low to moderate adherence to American Cancer Society guidelines for cancer survivorship, which includes diet adherence. Broader research by Esdaille et al. (2024) and McKinnon et al. (2014) shows that men with low SES are disproportionately exposed to unhealthy food options, high costs, and may have decreased knowledge surrounding healthy food choices. Cultural influences like family norms and faith also shape food choices and willingness to change (Brown, 2020; Rutledge et al., 2024). Culturally tailored dietary interventions have been shown to improve both adherence and satisfaction among racial and ethnic minority groups, especially when addressing both social support and outcome expectations (Cho et al., 2020; Vincze et al., 2021). These findings underscore the importance of identifying specific barriers faced by underserved men and adapting dietary interventions to meet their needs.

To our knowledge, no study has specifically looked at the barriers of adapting an MD to underserved Black and Hispanic men. To guide the development of future MD-based interventions, we sought to define general knowledge, perceptions and barriers to diet change and adherence in this population. We therefore performed focus groups to best determine barriers to dietary change and willingness to take part in MD-based interventions. Separate focus groups were done based on Harmon et al.’s (2015) study that provided evidence that dietary adherence differs between Black and Hispanic populations.

## Method

### Study Design and Participants

Following institutional review board’s approval, we employed a qualitative focus group design to explore knowledge, behaviors, and willingness to participate in MD-based dietary change. Participants were recruited at a single safety-net county hospital in Houston, Texas using purposive sampling. Participants who were seen in the urologic oncology clinic were approached via a combination of telephone and face-to-face visits. English-speaking Black or Spanish-speaking Hispanic White men between ages 30 and 75 years who were diagnosed with localized PCa within the last 2 years were eligible. In addition, given that health can be interdependent between partners, and that Black and Hispanic patients often rely on partners for help engaging in healthy behaviors (Bergner et al., 2018; Cho et al., 2020, 2025) spouses and significant others of any sex, gender, or race were eligible. Individuals were excluded if they had metastatic disease, were unwilling to take part in study procedures, or if they were diagnosed with PCa greater than 2 years prior to study enrollment.

A total of six focus groups, three in English, three in Spanish were conducted, each consisting of three to six participants to encourage discussion while maintaining manageability. Separate focus groups and analyses were performed for Black men (all in English) and Hispanic men (all in Spanish). Informed consent was obtained from all participants. Study participants underwent informed consent and were incentivized with a $50 gift card per participant. Focus groups were analyzed for thematic saturation, at which point further focus groups were not completed. Zero patients dropped out.

### Focus Group Procedures

**Figure 1.**
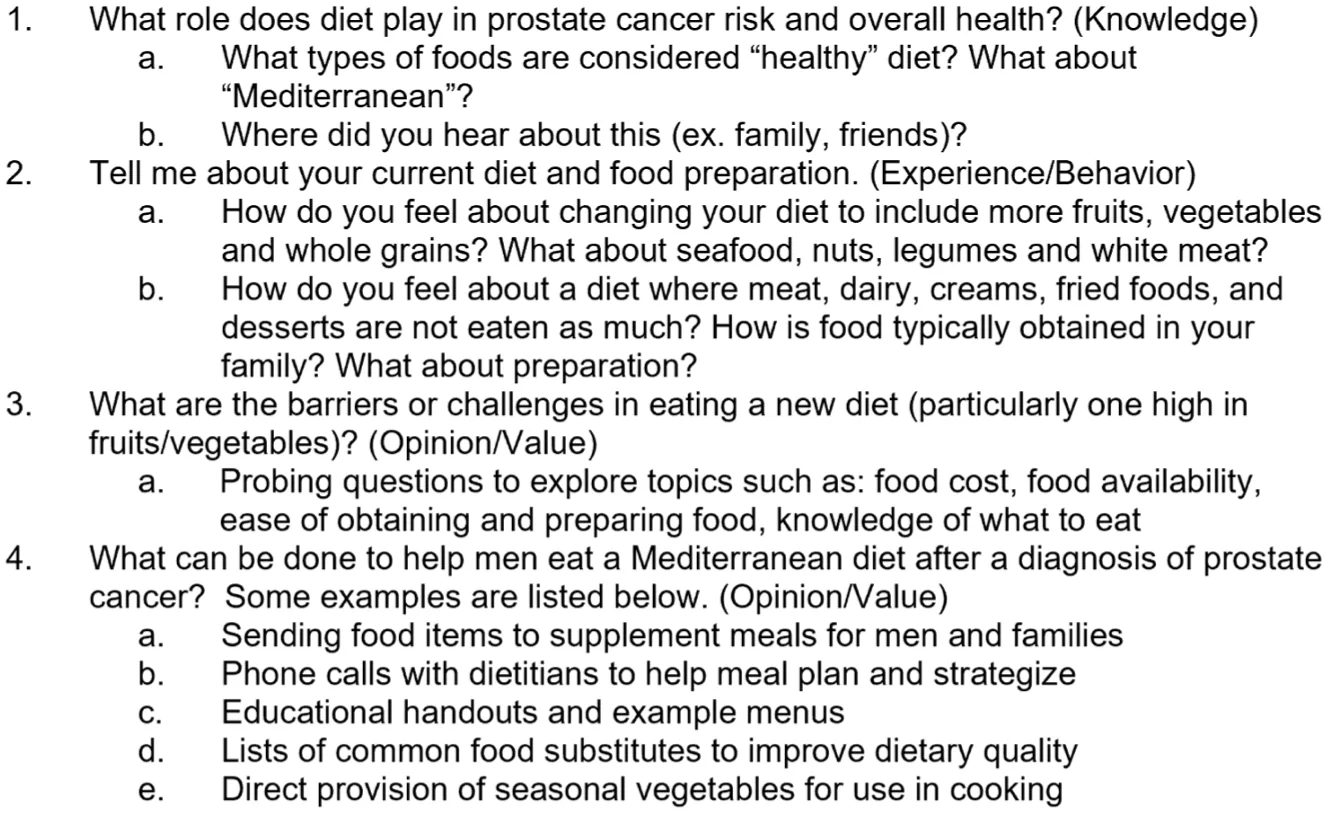
Outline of Focus Group Questions. Questions Are Designed to Address the Overall Question: “What Are the Barriers to Dietary Change Among Racial and Ethnic Minority Men With Localized Prostate Cancer at LBJ Hospital?” The Outline Consists of Four Main Questions (Question Type Denoted Parenthetically) With Follow-Up Questions Listed Below. These and Probing Questions Will be Used to Explore Responses.

Focus groups consisted of semi-structured, in-depth group interviews, conducted between November 2024 and March 2025 in person, at a single hospital. Each session lasted approximately 90 minutes. English sessions were moderated by one of two male physician scientists with experience in PCa, dietary intervention, behavior change, and qualitative research (J.R.G. and M.T.S.). Spanish sessions were moderated by a female trained senior coordinator with experience with qualitative research (M.T-E.). The Spanish-speaking moderator attended the English-speaking focus groups to attain knowledge on topics and questions. A physician was present at the Spanish-speaking sessions and two research coordinators were present at all six sessions. A semi-structured script was used to ensure consistency across groups while allowing for open-ended responses (Figure 1). Key topics explored included usual diet patterns, views of “healthy” versus “non-healthy” diet, knowledge of diet and PCa, and knowledge of the MD. Participants were made aware of the goals of the research before starting.

All focus groups were audio recorded with participant permission and transcribed verbatim with artificial intelligence software. Spanish focus groups were translated and then transcribed by GoTranscript. Field notes were also taken to capture nonverbal cues and group dynamics.

### Reflexivity

The researchers sought to maintain reflexivity throughout the study by frequently considering experiences and assumptions that could influence participants. Given the existing physician–patient relationship, special consideration was given to the development of the interview guide to ensure objectivity. Moderators explicitly acknowledged their role as facilitators rather than treating physicians and refrained from discussing individual health information. Questions related to specific treatments or medical appointments were gently redirected to private conversations after the focus group.

### Data Analysis

**Figure 2.**
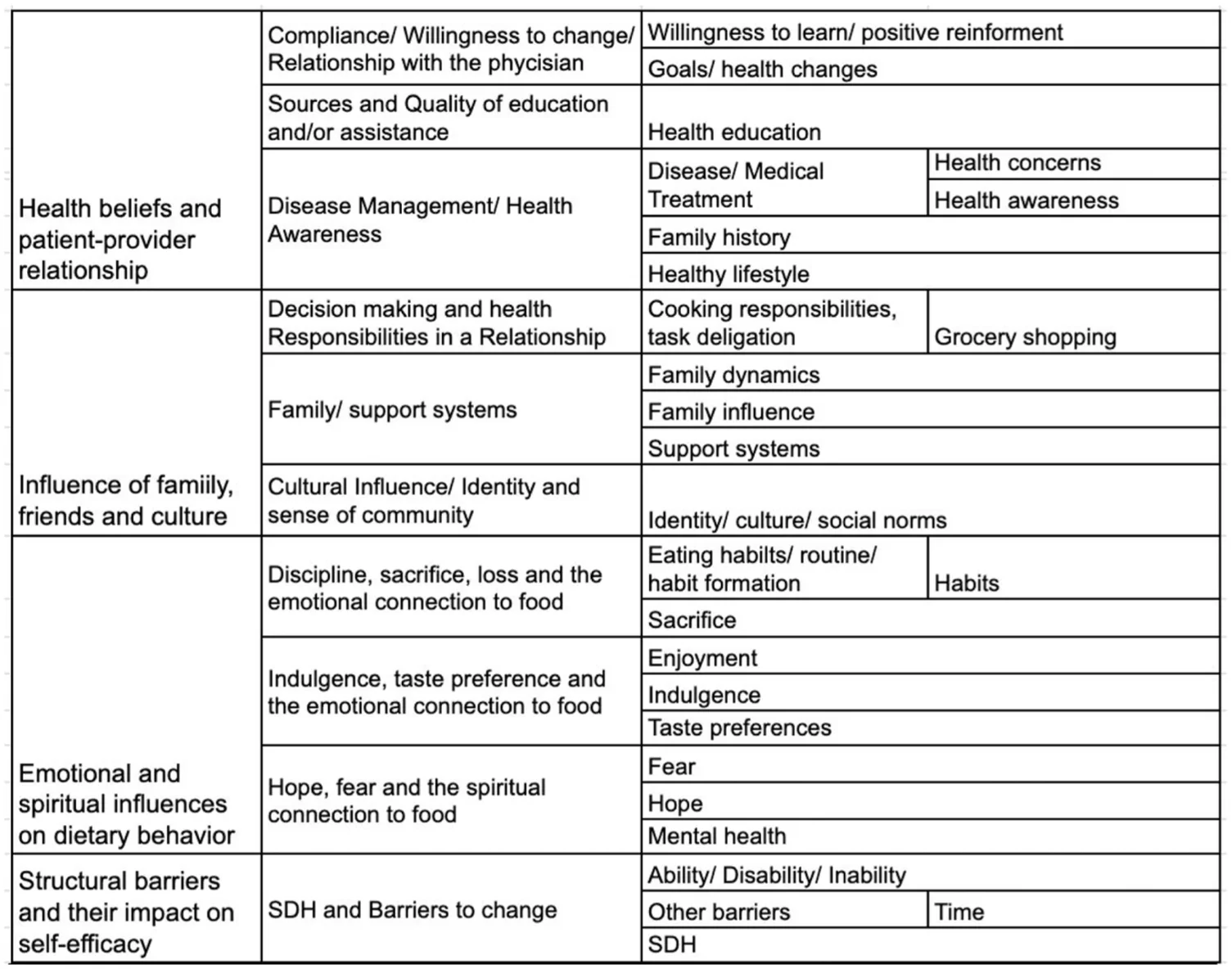
Coding Tree of Finalized Codes: Codes Were Structured Into Themes and Subthemes Based on Inductive and Deductive Coding.

Interview data were analyzed using thematic analysis using a hybrid framework based on constructs from the health belief model (HBM) and the theory of planned behavior (TPB) to guide our interpretation (Ajzen, 1991; Braun & Clark, 2006; Rosenstock, 1974). Deductive coding was initially employed to develop a preliminary codebook. Based on this coding, we hypothesized aspects from both theories would be influential (attitudes and subjective norms from TPB and perceived benefits and barriers from HBM). After conducting three focus groups, investigators reached consensus that data saturation had been achieved. Inductive coding was then used to refine and finalize a coding tree (Figure 2). We maintained an audit trail within ATLAS.ti web 8.7.0. This included importing and organizing transcripts, and revising the codebook based on inductive coding. Two independent coders (MTS and RSG) independently analyzed the transcripts to ensure reliability before coding. Discrepancies were resolved through discussion and consensus usually resulting in multiple codes per quotation. All coding decisions and revisions were documented within the software and the network view function facilitated theme development. Participant feedback on transcript content was not solicited.

## Results

A total of 108 men were screened, with 52 men approached and 18 men enrolled on the study. Demographic information from the focus groups is provided in Table 1 and Supplemental Table 1 .There were eight Black men with an average age of 60.8 (*SD* = 5.0) and 10 Hispanic men with an average age of 62.0 (*SD* = 4.8). While additional SES information was not collected, approximately 56% of patients served at this hospital are uninsured, highlighting lower income status. Thirteen men brought partners (five Black men and eight Hispanic men) while five attended by themselves. Our thematic analysis of the six focus groups led to the identification of four themes which did not differ between Hispanic and Black focus groups (though subtle differences in magnitude and subthemes were noted).

**Table 1.** Demographic Table.

	Total (*n* = 31)	Patients (*n* = 18)	Partners (*n* = 13)
Age ± *SD* (years)	60.4 ± 4.8	61.3 ± 4.7	59.1 ± 4.9
Gender			
Male % (*n*)	58.1 (18)	100 (18)	0 (0)
Female % (*n*)	41.9 (13)	0 (0)	100 (13)
Race			
Black % (*n*)	35.5 (11)	44.4 (8)	23.1 (3)
White % (*n*)	64.5 (20)	55.6 (10)	76.9 (10)
Ethnicity			
Hispanic % (*n*)	58.1 (18)	55.6 (10)	61.5 (8)
Non-Hispanic % (*n*)	41.9 (13)	44.4 (8)	38.5 (5)

Although this study was intentionally designed to explore the acceptability and adaptation of an MD-based intervention, we deliberately chose not to provide a full MD overview until after the focus groups to avoid biasing responses. Instead, the groups began with open-ended questions about participants’ definitions of healthy eating, barriers to dietary change, and current practices before discussing MD knowledge and interest (Figure 1). The MD overview, along with proposed culturally tailored substitutions, was introduced later in the session to allow for more authentic baseline responses. While most participants were broadly familiar with the idea of a Mediterranean-style diet, many lacked knowledge of its specific components and food items. As a result, participants’ quotes and resulting themes often appeared to reflect general dietary change. However, in context, participants were responding to questions framed within an MD-based intervention, and their feedback should be interpreted as reflecting attitudes and barriers relevant to MD adoption. Table 2 contains additional illustrative quotes categorized by subtheme.

**Table 2.** Illustrative Quotes Based on Themes and Subthemes.

Attitudes toward healthy diet and physician advice		
Subthemes	Hispanic	Black
Authority of doctor, compliance, and willingness to change	“He would also make green juices. I went to my doctor, and she scolded me.” She said, “Who told you that you could drink green juices? No, ma’am. Just because it’s trendy now to say, ‘Oh, I drink green juices’” She said, “It might be okay for her, but for you, who said so? Have you had your tests done? Have you had your analysis?” I said, “No.” She said, “Don’t take this the wrong way, . . . you Hispanics are quick to listen to your neighbor saying, “‘Try this, take that,” but don’t!” And she was right. I’m diabetic, and I’d throw all the greens and some ginger in, drink the juice, and feel so awful I’d have to sit down. Then my daughter asked, “What did you put in it?” And did some research. “Did you know ginger is really bad for you? . . . It lowers your sugar.” [partner]	So you got to be careful. The holidays are going to come up. Yeah. And I love a German chocolate cake. Oh, man. Man. I don’t know that the doctor would say [patient]
	I don’t drink alcohol. I haven’t felt like it in years. What I have eaten is meat, honestly. If they tell me, “This is harmful,” or “We need to replace it with fish or chicken,” then it has to be replaced [patient]	And I do like it, but we would cut it out, because not only do we want to live, we want to live a healthy life, so it wouldn’t be a problem, of course, you know, and we were talking about as you get older, sometimes things get, habits get harder to break, and we’ve been eating this cake for so long, and it might not be the easiest thing to do to stop it, but we would. [partner]
Sources and quality of education/assistance	No sugar because sugar helps those cells become more productive. No sugar, and try to follow a vegan diet with fruits and vegetables. That’s what my friends told me.[partner]	I think I’ve elevated my knowledge base since I found out about my friend, exponentially,. . .because now it’s close to home. . . . so we’re going to be more cognizant when we go back home. You know, they were saying, let’s not go here, let’s substitute the flaxseed for the butter or whatever. So it’s more of an awareness. And I think that’s the part that will get us over the hump, having the knowledge. I think that makes all the difference. [patient]
	From the internet, or, a lot of times, as Latinos, we–you know how we Latinos always carry our ancestors’ traditions with us—like, ‘Take this little tea for that.”[partner]	I’m thinking maybe if they would mail out more brochures to the potential prostate cancer candidates, or even have more of these type of settings, and get people in and sit down and talk about it, where the information is more, is put out there more than the doctors know it, the medical people know it, but the cancer patients don’t know this. [patient]
Disease management and health care awareness	My husband and I have always talked about it, but we haven’t actually put it 100% into practice. We haven’t said, “Today, we’ll completely change our diet. Today is the day.” We haven’t done it; we’ve always struggled with that process. Now, actually, I’ve told my husband, “Well, I think it’s time to take action and make this change we need,” because of his condition.[partner]	We used to eat rice or pasta like every single day in the past. But because of my high cholesterol, that’s when we make the changes, like with the rice and pasta and basically carbs. [patient]
	I also joined a weight loss program called [unintelligible]. They give you an injection to suppress appetite, and they’re teaching me to eat more nutritiously. If we eat meat, I pair it with stir-fried or raw vegetables.[partner]	Once I got diagnosed, I changed my diet.[patient]
The positive (and negative) influence of family, friends, and culture		
Subthemes	Hispanic	Black
Decision-making and health responsibilities within the relationship	[Moderator]: What about you? What do you do when your wife isn’t around? [patient]: I call her on the phone.[laughter] I was never interested in cooking. I even burn the water when I make coffee. She leaves me food when she has appointments. She tells me, “I left you food.” She has to act like I’m a little kid. She opens the fridge for me and says, “This is what you’re going to eat. I’ll be back at this hour.” Yes, but cooking? I never managed.	Well by me being the person that’s preparing the food at the house, I found that my brothers will eat just about whatever I fix any way I fix it. So I usually just fix it the way I like it. It’s easier for me . . . [But] Yeah. If it’s something that, uh, that my brother would eat. I think it will help. [patient]
	If I tell him, “My love, don’t eat this because it’s bad for you,” he listens. But when I’m not around, I don’t know what happens. [partner]	That’s what I try to do. [But] They want fried! And when they see me preparing chicken or when I tell them its chicken, they all heating up. Then when they find out its baked chicken they’re all like awwww. [patient]
Family/support systems	My daughters are very mature in that aspect because they take their dad to his appointments. The oldest one has been the most involved. She’s on board with anything that’s best for her dad. If we need to do something, she fully supports us, just like my other two kids. [partner]	My brother that did have a stroke, I don’t even monitor him no more because he’s kind of mind strong so he gonna do whatever he wants. But my other brother when I’m around him, we’re healthy, or at least what I think is healthy. And then my family at home, now, there are the ones that might be a problem because they have hamburger helper most of the time. And I think hamburger helper is pasta so that’s pasta all of the time. I have to stay away from that. And I’ll be trying to tell them about that. Because he grew up on noodles and he’s about 3 to 400 pounds and he goes back and forth to the doctor. He’s young, he’s about 28. [patient]
	I said, “No, dear, I’ll go. I’ll even follow it myself.” She said, “No, that’s just for my dad; you can’t do it.” I asked her, “Why?” “Because of your condition, you can’t follow the same diet as my dad, but you can pay attention, so you understand what he needs, buy what he needs, and cook for him.”[partner]	It’s hard for us to discipline ourselves to not eat certain foods because we grew up that way you know? [patient]
Cultural identity	My husband, because of where he’s from, eats hearty meals for breakfast, lunch, and dinner. [partner]	We have a Texas Southern University radio station and every Saturday morning they have a talk show and at least once a month they have a doctor on talking about prostate cancer for African American men and warning them and telling them to go and get checked at whatever age. And I think that’s good [patient]
	[partner] My routine from Monday to Friday is getting up at 4:30 AM and making tortillas on the stove, nice and warm. I pack lunch for my husband. Almost every day, I make him nopales with eggs and beans with eggs—what else do I pack? [patient] Meat once a week. [partner] Sopa aguada and stewed meat.	But I can say this, it’s hard, you know, as African Americans. Food. Eat food. Eat bad food. You know, we should, we should. start out young eating bad and so it takes sometimes things like this to happen to get a hold to our health when really we was not knowledgeable of how to eat young you know and then it’s not just an African American thing but it’s a human thing but I just know in my culture we came up eating a lot of foods [patient]
Emotional and spiritual influences on dietary behavior		
Subthemes	Hispanic	Black
Discipline, sacrifice, loss, and the emotional connection to food	But the truth is, I’ve made steamed vegetables for my husband sometimes, just vegetables. I told him, “This is how we should eat,” and he said, “Yes, but not always because it gets boring and tiring.”[partner]	So some things you just cannot do it because he have a weakness for food. And so it’s gonna be, you know, you gotta stop buying. [patient]
	[Moderator] “I can cheat here and there.” What do you think about that? What is your understanding of cheating here and there? [partner] It wouldn’t be right because we would only be fooling ourselves. We are following a diet, so why cheat? [patient] You’re speaking the truth.	And that kind of helps the discipline side, because if you don’t have it in the house, you got to find other alternative things that you wouldn’t mind eating . . .That’s not harmful to your appetite. . .not harmful to your health. Because when you bring it home, you know, you’re going to cook it . . . you gotta find some food that’s an alternate that would make you happy, you know, in spite of eating the food you’re trying to give up [patient]
Indulgence, taste preference and the emotional connection to food	When I have the urge to drink Coca-Cola, I drink it—that’s not a problem.[partner]	I do know my main area that I have to focus on is staying away from the sugar, because I like cake and cookies and pies, and I’ll indulge in that in a heartbeat. Like the other day I told [my mother] I wanted a pineapple upside down cake, so she bought one. But instead of having one quarter of it, which is what I would normally take, I just took a small slice, and that will last me a few days now, versus five years ago I would have eaten the whole thing in two days. [patient]
	I can’t give it up. It’s a battle, but I always have some [partner]	Ham hock, collard greens, canned meat, pork chops . . . lamb and veal pork chops smothered in cream gravy. Oh, chicken wings, turkey wings . . . Soul food! Boudin sausage. And you know, [I] just eat like a hog. That’s what [I[ does. Every time I cook, I cook so much, she freezes it so I can go back, you know, around [to] it. Yeah, but I’m 5’6”, and I weigh 210 pounds, you know, and I’m gaining weight instead of losing it, so I think this is why God, this is why God had me up inside my house . . .[when] Jose called me [about this study] because we got a situation. [patient]
Hope, fear, and the spiritual connection to food	I also read that, but I’ve put my faith in God. [partner]	Do it now versus later. You know, laying up in a hospital bed and they’re telling you, well, this is what’s got to happen now in order for you to live another six months. . . You know, right now we’re at a point where we’re good, you know, because [you can], you know, get hit by a train or something, God forbid. Yeah. I have control over some parts of my life in terms of I can exercise, I can eat properly, take my medication, hopefully live a long time. [patient]
	If you keep feeding me this, I will die [patient]	I have first-hand experience with someone that passed away early because of prostate cancer, so that was like a slap in the face. [patient]
Structural barriers and their impact on self-efficacy		
Subthemes	Hispanic	Black
Cost	We buy generic stuff. We don’t buy special foods for the little kids like we used to. “No, he eats this.” Nowadays, we don’t. My husband will start working soon, so it shouldn’t be a challenge, but right now, it is because we have to manage with what our son or daughter can bring us, but they also have their expenses. So, right now, it is. [partner]	I don’t get to get out and make I don’t because I don’t have enough for groceries. I’m like . . . I mean, but I don’t have a steady income for food. So, if I have enough for a burger, I’m getting burger and fries. [patient]
	Everything is more expensive when you’re trying to eat healthier. However, getting fat is quite cheap. [partner]	I would love to do it but I can’t. I never can’t afford nothing like that. And how can [I]? I would love getting salad and stuff every single day. I just can’t afford it.. . . As that type of food is expensive. The cheap food is the good food . . . All the stuff, the bland stuff. I can’t afford. Yeah. [patient]
Other barriers	[I] don’t know how to cook the Mediterranean diet [partner]	They actually do give natural stuff to us, well to me, but when I get it, the box is like two years old. The vegetables are almost old. You can’t really eat them. Some of it you had to cut off part of it because it’s already rotten. And then the rest is starch. And I’m not gonna eat the starchy like the potatoes and stuff. . .I can’t eat that . . . And even the fruit will be almost spoiled . . .two or three days you better throw it away. [patient]
	I think the challenge would be everyday. Since I’m alone . . . [patient]	I can’t eat the salad. Because I can’t chew it. [patient]

### Theme 1: Attitudes Toward Healthy Diet and Physician Advice

One goal of the focus groups was to determine the quality and sources of information regarding diet and attitudes toward diet change. The definition of a healthy diet was relatively accurate although sometimes extreme (completely plant-based). There were also some perceived myths. One Black patient noted “I’m doing bad. You supposed to have a glass milk a day” with another Black patient saying, “You don’t [supposed to] have any type of chicken, you know, just salad.” There was also some confusion regarding overall basics of nutrition. One Black participant stated, “I don’t eat red meat. I don’t eat no kind of red meat.” but later discussed a typical breakfast of pork bacon and sausage. Apart from one partner of a Black patient being from the country of Turkey, most participants could not name more than a few aspects of the MD. At the end, when asked how the intervention could be improved, many participants asked for even more instruction and stated the inclusion of a nutritionist “would be essential” (Hispanic patient).

Presence of comorbidities also played a large role in perceived risk and attitudes. “But we always are cognizant of the fact that we’re diabetics, and so we’ve got to have some green vegetables, not just green vegetables, but different colors, sweet potatoes, carrots.” (partner of a Black patient). High cholesterol, bariatric surgery, and HIV were other medical conditions mentioned. The PCa diagnosis and treatment itself was also a major cue to action for multiple men and their partners. One Black patient and one partner of a Hispanic patient stated, “Once I got diagnosed, I changed my diet” and “Ever since they told my husband he had cancer, I’ve basically got rid of it [meat].” Overall participants were very receptive to the idea that a healthy diet could be beneficial for PCa and willing to do “whatever will help” (partner of a Hispanic patient).

Most information came from the internet or friends and family. Doctors were generally seen as the final authority in terms of nutrition and had the power to override other information. “And we do trust more what the doctors say. Not more. That’s the only thing we trust” (partner of a Black patient). One partner in the Hispanic group mentioned “I gather information . . . I always pay attention because I’m interested in what they’re saying. If I’m on the internet or see a family member who is a doctor, I ask them questions.” Other sources of information include “reading in brochures, looking it up, googling it” (Partner of a Black patient).

### Theme 2: The Positive (and Negative) Influence of Family, Friends, and Culture

Food is a major aspect of culture. Here the subjective norms within the TPB shine as major influencers of perceived behavioral control and self-efficacy. As expected, partners/spouses are critical in support and diet adherence. Grocery shopping and cooking responsibilities fell on the partner in almost all of the coupled men. Men with partners who were pushing them to eat healthier expressed gratitude, and the partners seemed to take pride in this role.

For some subjects, extended family and friends were also perceived as members of the support team. Here, Hispanic adult daughters were especially well represented as policing food choices and ensuring patients made medical appointments. One partner exclaimed: “ No, they didn’t tell me anything. She [her daughter] told me, “Go and ask them what he can or can’t eat.” In one of the Black focus groups, one patient cited his mother as a positive resource “Well, I have a good source at home, my mother . . . she’s a diabetic too . . . But she’s a good source for different things when it comes to food.”

Other participants named family members as perceived barriers. Here family members can serve as sources of temptation or discouragement. “An obstacle for me would be when my daughters visit us. They say, “Mom, look what I brought. Look, my love.” In those cases, I can’t say to my daughters, “Oh, my love. I can’t eat this, sweetie.” They bring those things to us with a lot of love, so we should say, “Yes, my love. All right. I’ll have a little.” That would be somewhat of a barrier for me. . . . I can’t. I don’t feel well. My heart doesn’t feel good if I don’t accept what my daughters bring me” (partner of a Hispanic patient). Familial responsibilities can also be challenges. One family has a son with autistic spectrum disorder and his diet is extremely limited. Another patient has two brothers staying with him, one of which is disabled. Any diet adjustment would require accounting for these family members.

In general culture was seen as a source of pride but a barrier in diet change. Both groups spoke fondly about staple foods in their culture with nods and agreement from other group members. A Hispanic patient joked: “Because of our customs, the habits we carry. You can’t say no to a barbecue. Everyone surrenders to a barbecue or a stuffed arepa. [group laughter].”

### Theme 3: Emotional and Spiritual Influences on Dietary Behavior

In addition to cultural connections, the relationship to food is extremely personal. Interestingly, the way indulgences were expressed changed based on the focus group environment. In groups where there was negative view on the difficulty of dieting, indulgence was almost a source of pride. “I think if I want to cheat, I can cheat. . .I’d rather do it openly than in secret . . .” (partner of a Hispanic patient). But in groups where one or two participants were trying to maintain a healthy diet, talking about indulgences resembled confessions. “That’s kind of a trigger . . . You can go for a while without some of that stuff. Yeah, all of a sudden, . . .you got to just taste it . . . I mean, I can stop but I just got to just have it just for a moment” (Black patient).

In addition, the attitude toward the proposal of a diet change can lead to feelings of loss and sacrifice. A despondent Hispanic patient mentioned “I’ve heard bread is bad. I like bread.” In a moment of levity, one Black patient looked at the snacks we provided for the focus group (Greek yogurt with granola, fresh fruit, assorted nuts, etc.) and said “so what’s wrong with fasting? because if you gotta eat THAT, I just won’t eat.”

Noting the intense emotional response from discussing food, it’s not surprising that participants turned to spiritual connections for support. This is one place where the two groups diverged. Both groups spoke on religion and spirituality, but the Hispanic groups had a stronger connection between their spirituality and their diet.

I have a lot of faith in God, so I kept praying. “Lord, I know you will heal him. With your help, everything I do—all the remedies—will work through your hands.” I gave him natural remedies for three months, and when they did another biopsy, they said the cancer had improved. (Partner of a Hispanic patient)

Black participants also displayed spirituality, but it was more generalized and often fearful. Some examples include “I’m trying to stay away from here [the hospital] . . . because I’m trying to fight food [and] fighting the disease [and] you’re losing the battle because the food always gonna win” (Black patient).

### Theme 4: Structural Barriers and Their Impact on Self-Efficacy

Acknowledgment of economic burden is critical in any diet-based intervention. The most cited perceived barrier was cost. Cost affected meal choices, quantity, quality, and frequency of meals. “We eat a lot of pork because it’s cheaper” (partner of a Hispanic patient). Even in situations where the participant was willing to make changes, the food had to be “something that I can afford . . . especially [if it’s] the doctor who recommended it” (Black patient).

Another barrier was their ability to create meals and knowledge of Mediterranean recipes. One partner of a Hispanic patient mentioned,

I know about this and that, it’s healthier, but I don’t know how to prepare it. Another Black patient stated “I would rather have a class that teaches me how to put it together . . . I want to know how to prepare the food . . . Now I think that would help me out a whole lot.

**Figure 3.**
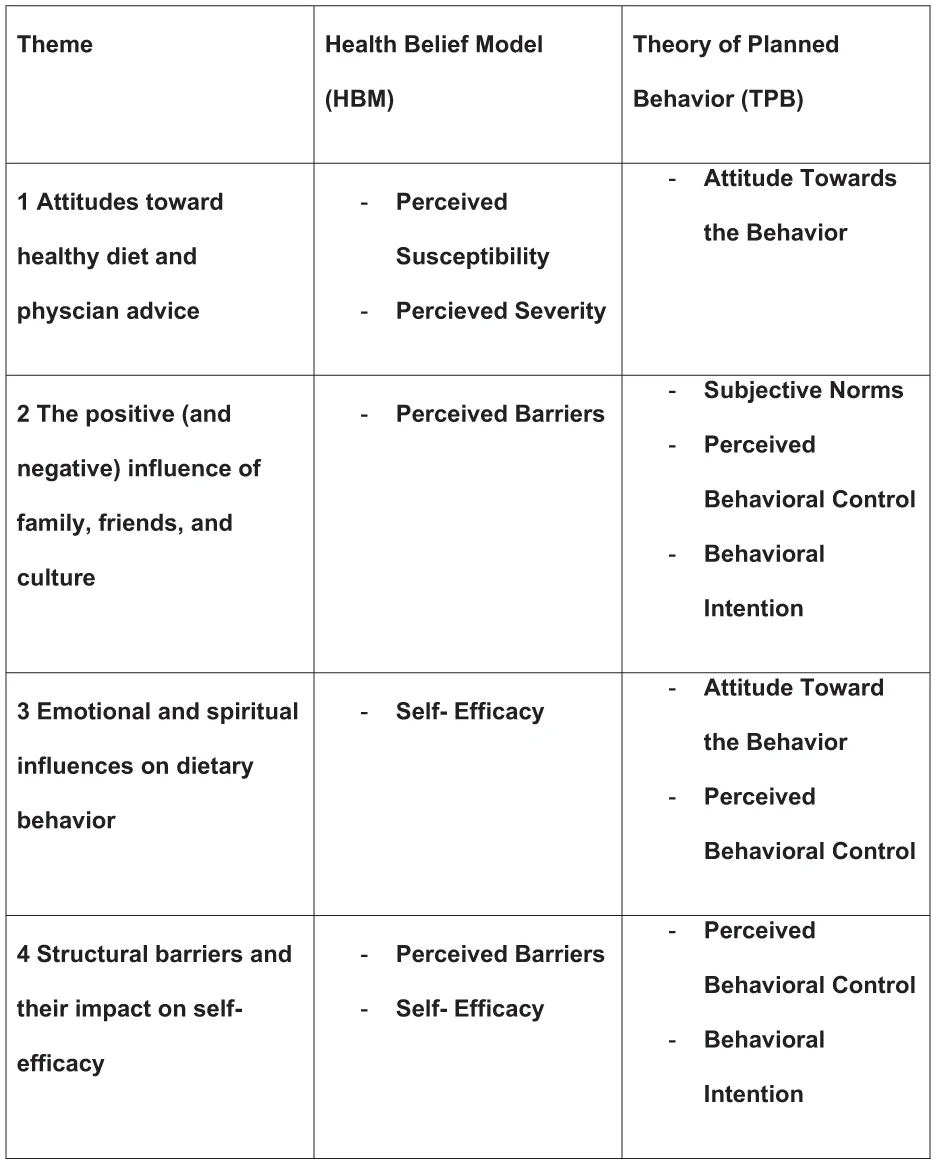
Conceptual Diagram Linking Constructs to Themes.

Figure 3 demonstrates connections between themes and HBM and TPB model constructs. Theme 1: Attitudes toward healthy diet and physician advice, explores the constructs of perceived susceptibility, perceived severity, and attitude toward behavior. Theme 2: The positive and negative influence of family, friends, and culture, explores the constructs of subjective norms, perceived behavioral control, behavioral intention, as well as perceived barriers. Theme 3: Emotional and spiritual influences on dietary behavior, explores attitudes toward behaviors, perceived behavioral control, and self-efficacy. Finally, Theme 4 focuses on structural barriers and their impact on self-efficacy, perceived behavioral control, behavioral intention, and other perceived barriers.

## Discussion

### Principle Findings

This study sought to describe dietary patterns and barriers to dietary change among underrepresented racial and ethnic minority men with PCa and their partners. These data will help inform future behavioral intervention development, including the use of MD-based interventions among medically underserved Black and Hispanic men. Interestingly, while focus groups were completed separately, themes that emerged were consistent among Black and Hispanic patients. This included noting the importance of physician opinions and recommendations, emotion and spiritual aspect of eating, the role of structural barriers (mostly related to cost), and friend and family support. Our results suggest a few key aspects that must be prioritized in the design of future intervention.

Baseline knowledge can be misaligned and certain myths and assumptions should be addressed early. Participants need clear and accurate nutritional education including fundamental knowledge and direct recommendations with recipe support. Participants also felt participation of someone with nutritional expertise was crucial. Formal education, especially by dietitians, can address perceived benefits and barriers, and influence self-efficacy. Source of information is important and advice from physicians can override other sources and serve as a powerful cue to action.

Family members can serve as important allies and/or obstacles to dietary change and buy-in is vital. Special attention needs to be given to family members who pursue simultaneous dietary change. Partners and spouses of the men are critical and will often take the lead on interventions. Adult children are also important in maintaining consistency and evaluating new foods. Early and intentional buy-in from key members can align subjective norms in favor of the intervention, particularly given the importance of family support that were especially highlighted among Hispanic participants.

Any intervention must include space for the emotional and, at times, spiritual aspect of food and culture. Feelings of joy, loss, sacrifice, and shame can accompany foods and dieting attempts and strongly influence attitudes toward behaviors. Allowing for social interaction and community support between participants may alleviate pressure. This opens up opportunities for community partnerships (local churches, faith-based organizations, and social groups)

Finally, interventions must account for economic burden. Attention to finances weaved through every aspect of the focus groups and will be one of the largest barriers to dietary change. Also, participants’ ability to cook and eat the meals must be examined. The idea of affordable meal preparation and cooking classes was well received. Table 3 outlines how themes can be implemented into an MD-based intervention. We note that our conclusions are in line with prior studies. A systematic review by Tsofliou et al. (2022) of MD adherence in adults (not limited to cancer patients or medically underserved) listed lack of education and recipes, cost, negative influence of family, cultural differences, and more as barriers. Facilitators included family/friend support and disease prevention. Siapno et al. (2024) performed a study looking at low-income men and general health behavior change found cost as a major barrier and strong support systems as the largest facilitator.

**Table 3.** Practice Implementation Keys Based on Themes.

Theme	Implementation
1. Attitudes toward healthy diet and physician advice	• Provide clear, accurate nutritional education with recipes • Include a dietitian • Address myths and build self-efficacy • Leverage physician advice and authority as a cue to action
2. Influence of family, friends, and culture	• Engage partners, spouses, and adult children early • Ensure their buy-in to align subjective norms • Recognize family as both support and potential barriers
3. Emotional and spiritual influences on dietary behavior	• Acknowledge emotional and spiritual ties to food • Create space for community support, including engagement with spiritual organizations • Allow for social interaction to ease pressure
4. Structural barriers and self-efficacy	• Address financial limitations • Ensure meal affordability • Assess cooking skills and access to resources including culinary classes • Reduce practical barriers to dietary adherence

### Strengths and Limitations

Strengths of the study include the combination of English and Spanish focus groups which allow inclusivity often missed in similar studies. Due to the similarities between the two groups, English-speaking Hispanic focus groups were not needed, especially considering most Hispanic patients seen at this institution speak Spanish. Our use of bilingual study personnel enhanced familiarity and comfort and performing separate focus groups (and analyses) enabled our group to highlight nuances surrounding food and culture.

There were several limitations in our study. Each of the six focus groups were limited to eight participants (four patients and up to four spouses), which, while somewhat limiting sample size, allowed for individual perspectives and for the study team to observe couples’ interactions. We also note that saturation was met after 3 focus groups for each demographic. However, further studies in expanded populations are needed to determine if these findings are generalizable to other medically underserved groups In addition, there is some risk of self-selection bias, whereby interested participants may have different experiences (and question responses) than those who chose not to participate. Finally, it is not clear how structural racism, which likely affects many of the barriers noted in our analysis (Aaron & Stanford, 2022) would ultimately impact the design and execution of behavioral interventions focused on dietary change in these groups. To summarize, all the men (and their partners) were interested in an intervention that prioritized their needs and focused on improving their health.

## Conclusion

This study sought to qualitatively determine the patient’s and significant other’s knowledge about the MD, baseline diet, barriers to dietary change, and specific feedback on an MD intervention. Despite differing cultures and experiences, we found that participants were willing to participate in an MD-focused intervention. This study provides preliminary evidence that MD-based interventions are feasible in this population and participants are amenable to the change. Further implications for practice include key focus points for a successful diet-based intervention.

## Supplemental Material

sj-docx-1-heb-10.1177_10901981261421578 – Supplemental material for Determining Barriers to Dietary Change Among Medically Underserved Men With Prostate Cancer Supplemental material, sj-docx-1-heb-10.1177_10901981261421578 for Determining Barriers to Dietary Change Among Medically Underserved Men With Prostate Cancer by M. Smith, R. S. Garcia, D. Cho, M. Tariba-Edick, T. Lawen, C. Daniel, L. McNeill, C. Pettaway and J. R. Gregg in Health Education & Behavior
