# Supplementary material for: Determining Barriers to Dietary Change Among Medically Underserved Men With Prostate Cancer
Source: Health Educ Behav. 2026 Feb 25;53(5):501–12. doi: 10.1177/10901981261421578 (PMC13140757; doi:10.1177/10901981261421578)
Supplement: Supplementary file 1 [file sj-docx-1-heb-10.1177_10901981261421578.docx]

| **OnCore #** | **Participant** | **Sex** | **Ethnicity** | **Race** |
| --- | --- | --- | --- | --- |
| 1 | Patient | Male | Non-Hispanic | Black or African American |
| 2 | Patient | Male | Non-Hispanic | Black or African American |
| 3 | Spouse | Female | Non-Hispanic | Black or African American |
| 4 | Spouse | Female | Non-Hispanic | White |
| 5 | Patient | Male | Hispanic or Latino | White |
| 6 | Spouse | Female | Hispanic or Latino | White |
| 7 | Patient | Male | Hispanic or Latino | Unknown |
| 8 | Patient | Male | Hispanic or Latino | White |
| 9 | Spouse | Female | Hispanic or Latino | White |
| 10 | Patient | Male | Hispanic or Latino | White |
| 11 | Spouse | Female | Hispanic or Latino | White |
| 12 | Patient | Male | Non-Hispanic | Black or African American |
| 13 | Patient | Male | Non-Hispanic | Black or African American |
| 14 | Patient | Male | Non-Hispanic | Black or African American |
| 15 | Patient | Male | Hispanic or Latino | White |
| 16 | Spouse | Female | Hispanic or Latino | White |
| 17 | Patient | Male | Hispanic or Latino | White |
| 18 | Spouse | Female | Hispanic or Latino | White |
| 19 | Patient | Male | Hispanic or Latino | White |
| 20 | Spouse | Female | Hispanic or Latino | White |
| 21 | Patient | Male | Non-Hispanic | Black or African American |
| 22 | Patient | Male | Non-Hispanic | Black or African American |
| 23 | Patient | Male | Non-Hispanic | Black or African American |
| 24 | Spouse | Female | Non-Hispanic | Black or African American |
| 25 | Spouse | Female | Non-Hispanic | Black or African American |
| 26 | Spouse | Female | Unknown | White |
| 27 | Patient | Male | Hispanic or Latino | White |
| 28 | Spouse | Female | Hispanic or Latino | White |
| 29 | Patient | Male | Hispanic or Latino | White |
| 30 | Spouse | Female | Hispanic or Latino | White |
| 31 | Patient | Male | Hispanic or Latino | Unknown |

**Supplementary Table 1: Patient and Partner Demographics**
